# The effect of surface roughness on capillary rise in micro-grooves

**DOI:** 10.1038/s41598-022-19111-w

**Published:** 2022-09-01

**Authors:** Gholamreza Bamorovat Abadi, Majid Bahrami

**Affiliations:** Laboratory for Alternative Energy Conversion (LAEC), School of Mechatronic Systems Engineering, Simon Fraser University, Surrey, BC V3T 0A3 Canada

**Keywords:** Engineering, Physics

## Abstract

The capillary action is a unique feature of micro-grooves with numerous applications. This spontaneous flow eliminates the need for an extra pumping device to deliver a liquid. Capillary action depends on physical properties and features of the solid surface, as well as on thermophysical properties of the liquid. In this study, our previously proposed unifying capillary rise model is extended to include the effect of surface roughness. A new characteristic length scale is proposed that includes salient geometrical parameters, such as micro-grooves height, width, and surface roughness. Furthermore, it is shown that by using the proposed characteristic length scale, it can be determined whether the capillary action would occur in a given micro-groove and liquid. Various metallic and polymeric surfaces with a wide range of surface roughness are fabricated from aluminum, stainless-steel, natural graphite sheet, and 3D-printed stainless-steel and a polymer. A profilometer and sessile drop method are used to measure surface roughness and the contact angles, respectively. The present unifying model is compared against our measured data, and it is shown that it can predict the capillary rise in rough micro-grooves with less than a 10% relative difference. It is observed that the capillary height can be increased for a wetting surface by introducing surface roughness and by using optimal micro-groove cross-sections that are triangular as opposed to rectangular. The proposed compact, unifying model can be used to predict the capillary rise for any given micro-groove cross-section, and as a design tool for numerous industrial and biomedical applications, such as heat pipes, power electronic cooling solutions, sorption systems, medicine delivery devices, and microfluidics that utilize capillary micro-grooves.

## Introduction

The self-driving flow of a liquid in a capillary micro-groove has a wide range of applications, such as in space engineering due to microgravity, in power electronics and heat pipes, and in sorption technology and capillary-assisted evaporators. One major application of capillary action is in the micro-grooves of heat pipes. Heat pipes, as a two-phase heat transfer device, are crucial in the design of many power electronic devices^1–4^. A general understanding of flow in open micro-grooves and its limitation is also available in the literature for different geometries^5–11^. In sorption cooling and heat pump technology, the main obstacle preventing commercialization is size and weight. Capillary-assisted low-pressure evaporators (CALPEs) are used in closed-cycle sorption systems, including heat pumps, heat transformers, desalination, and thermal energy storage systems as a compact solution^12^.

In our previous study^13^, a new unifying analytical model was proposed to predict the capillary rise in smooth micro-grooves with a wide range of cross-section geometries as a function of: (i) contact angle, and (ii) a novel characteristic length scale of the micro-grooves, defined as the ratio of the liquid–vapor to the solid–liquid interface, i.e., $h^{*} = cos\theta - \frac{W}{{P_{w} }}$.

In the present study, the effect of surface roughness and its impact on contact angle is investigated and added to our unifying model and experimentally validated. Surface roughness exists in all real and engineered surfaces, especially 3D printed and etched substrates.

The capillary action has long been the focus of much research. Leonardo da Vinci might have recorded the first observation of the capillary phenomenon^14^. Years later, Robert Boyle performed experiments by inserting a capillary tube in red wine and observing the independence of the liquid column from the pressure on top of the column^15^. The capillary action was successfully quantified in 1805 by Thomas Young and Pierre-Simon Laplace; i.e., the well-known Young–Laplace equation for capillary action^16^. Albert Einstein published his first paper on capillarity^17^.

Recently, researchers have investigated capillary rise under numerous conditions, including zero gravity, tilted tubes, non-circular conduits, tubes with rough surfaces, and porous media^18–22^. Wang et al.^23^ studied the capillarity rise in micro-grooves with rectangular cross-sections analytically and experimentally. They used a Helmholtz free energy method to model the capillary rise in a vertical open micro-groove and experimented with micro-grooves made by photolithography. They reported the effect of the micro-grooves’ width on the capillary rise. Chen^5^ studied the flow of a wetting fluid in metallic micro-grooves with various depths. They concluded that the flow in micro-grooves was proportional to the square root of time. Khumpuang et al.^24^ modeled the capillary rise in a quadruplets-microneedle made by X-ray lithography for blood extraction. They compared their modeling results with experimental data and reported a good agreement. Yang et al.^25^ studied the dynamic flow of capillary phenomenon by using a water and water–glycol mixture in hydrophilic micro-grooves. They reported that the capillary rise was faster in a micro-groove with a smaller width, regardless of its geometrical cross-section. Extrand^22^ studied the forces, pressures, energies and kinetics of capillary rise in chemically homogeneous tubes and tubes with chemical gradients. Focusing on heat pipes, Wu et al.^26^ explored the potential of increasing the capillary force in grooved wicks by utilizing a novel skew-grooved structure when compared with a rectangle-grooved wick. They reported an improvement in capillary force by using a new structure as opposed to a rectangular micro-groove.

Contact angle depends on the specific solid–liquid interaction, environmental conditions, such as temperature and humidity, and surface features such as roughness. Therefore, many researchers attempted to quantify this interaction by experimental means. Smith et al.^27^ studied the wettability of a fluid–solid interface with an application in oscillating heat pipes. They used two techniques to measure the contact angle: (i) the sessile drop method, and (ii) capillary rise. Reported contact angles included the interaction between copper, aluminum, and Teflon with liquid water, acetone, R-134a, and HFO-1234yf. Schwartz^28^ provided a molecular interpretation of contact angle and studied an intrinsic contact angle, defined as variations not related to surface roughness, heterogeneity, or penetrability of the solid surface. Tadmor and Yadav^29^ showed that the as-placed contact angle of a droplet decreased with the droplet size since its hydrostatic pressure increased. Khandekar et al.^30^ studied the contact angle in pulsating heat pipes made from real engineering surfaces, as well as ideal smooth surfaces. Diaz et al.^31^ hypothesized that the adsorption of liquid film in the droplet vicinity was the reason for intrinsic hysteresis during the sessile drop measurement of static contact angles. Butt et al.^32^ defined the boundary conditions of Young’s equation and then showed the effect of evaporation for macroscopic droplets to be negligible. Rodríguez-Valverde et al.^33^ proposed a model for predicting the Young contact angle of rough solid surfaces based on the contact angle hysteresis measurements. Tadmor^34^ reported the line energy to the contact angle resulted from the surface imperfections. Tadmor’s proposed relationship was a function of the droplet volume, the interfacial energies, and the measured contact angle. Lamour et al.^35^ proposed a simplified experimental setup to measure the contact angle as opposed to commercial goniometers and argued that their setup was precise enough for most applications, easier to construct, and affordable. Bernardin et al.^36^ showed the temperature dependence of water-aluminum’s contact angle, experimentally. They reported that for temperatures below 120 °C, the contact angle remained unaffected. Others have studied the contact angle theoretically, and experimentally^37–39^.

Wenzel^40^ first proposed that the wetting properties of a solid surface should be directly related to surface roughness. He concluded that the increase in the surface area of fibrous materials plays a large role in the hydrophobicity of the tested samples. Later, Cassie and Baxter^41^ extended Wenzel’s relationship between roughness and contact angle and applied it to porous surfaces. They contributed some naturally occurring hydrophobicity, such as in duck feathers to its unique structure. Tamai and Aratani^42^ studied the effect of surface roughness on the contact angle for a silica glass-mercury interface using a sessile drop method. They concluded that Wenzel’s model holds for their experiments, showing the variation in contact angle of silica glass samples with various roughness ratios. Ryan and Poduska^43^ developed an experimental method to show the effects of surface roughness on contact angle on solid surfaces. They showed that the change in surface energy due to surface roughening was responsible for the change in the contact angle. Berim and Ruckenstein^44^ calculated the microscopic contact angle of a liquid droplet on a rough surface. They recognized two limiting cases: (i) Wenzel, and (ii) Cassie-Baxter regime. Li et al.^45^ studied samples with various surface roughness and used phase-field interface tracking to simulate the wetting phenomenon. They concluded that when the roughness increased, the contact angle of a hydrophilic surface would decrease.

The effect of geometry on capillary meniscus is studied in the literature for limited cases. Concus and Finn^46^ provided an estimate for the height of the equilibrium meniscus in a wedge with interior angle *2α*, for a case when a liquid partially fills a cylindrical container. They showed that the qualitative behavior of such a surface, changes near the vertex, and depending on the contact angle and *2α*, the surface is either bounded or unbounded. Borhan et al.^47^ studied the capillary rise of a liquid between two sinusoidally corrugated plates. They concluded that the spatial variations in the capillary height at the centerline are negligible while those closer to the wall follow the variations in the capillary cross section. They observed a relative enhancement in the centerline capillary height as compared with the capillary height in a parallel plate. Bico and Quéré^48^ discussed the capillary rise of a liquid inside an angular capillary tube and showed that for a wetting liquid, the capillary height is inversely proportional to the length of confinement. Hill and Pozrikidis^49^ studied the hydrostatic meniscus shape in a vertical plate or circular cylinder with periodic corrugations. They discussed the effect of wall irregularities on the shape of the contact line and vertical component of the capillary force by providing analytical and numerical analyses.

Although the capillary phenomenon and the parameters affecting it are studied, a comprehensive capillary rise model that includes the surface roughness effect has not been proposed. In this study, a new closed-form and unifying capillary rise model is proposed that includes the effect of surface roughness. The proposed model is compared with our experimental data in micro-grooves with rectangular and triangular cross-sections. The effects of the micro-grooves’ height, width, contact angle, and surface roughness are experimentally investigated and reported.

## Model development and the effect of surface roughness

**Figure 1:**
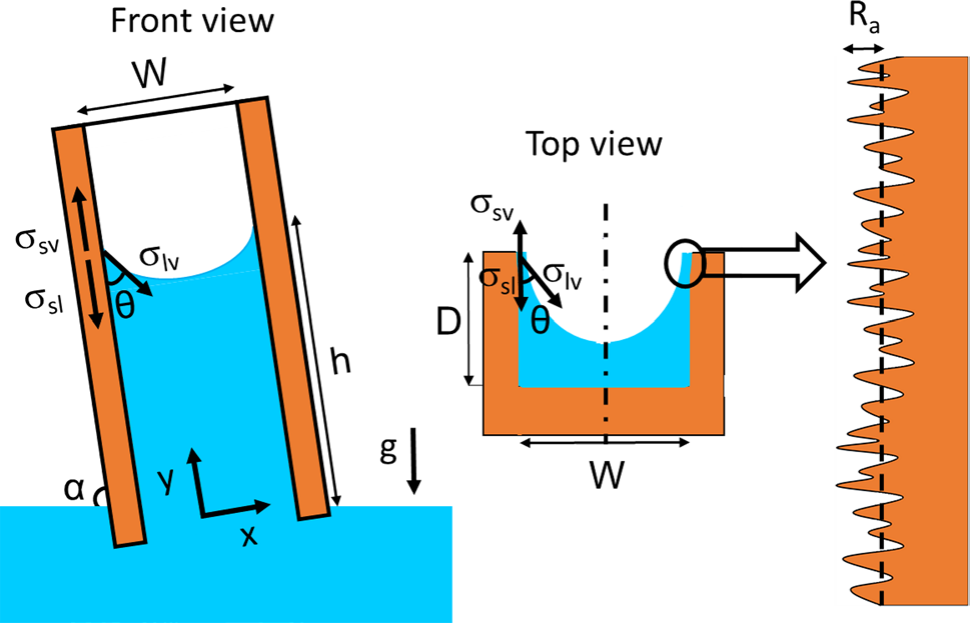
Top and front view of capillary rise, *h*, in a rough micro-groove with width of *W* and depth of *D*. Our previous model^13^ is extended to include the effects of surface roughness. Dash line shows the mean surface line. *α* denotes an inclination angle of the capillary channel.

We start with a micro-groove with a rectangular cross-section, see Fig. 1. When a micro-groove is placed in a liquid as shown in Fig. 1, the liquid rises along the micro-groove due to capillary forces. The governing equations, main assumptions, and the solution approach to predict the effect of surface roughness on capillary rise are listed in this section. Similar steps can be taken for any other cross-section. The present model assumptions are as follows:

- The open micro-grooves’ width is small enough for the capillary action to occur (10 nm < length scale < 1 cm)^50^,
- The surface roughness has a Gaussian (random) distribution, which is created in our surfaces using an abrasive polishing method,
- The surface is wetting, or hydrophilic,
- The micro-grooves are filled with the liquid,
- The micro-groove is placed with a slanted angle, alpha, and that its bottom end always touches a fluid reservoir,
- The physical properties are constant,
- The vapor–liquid interface is homogenous, and
- Heat transfer is negligible since the capillary action is a fast, almost instantaneous process.

**Figure 2:**
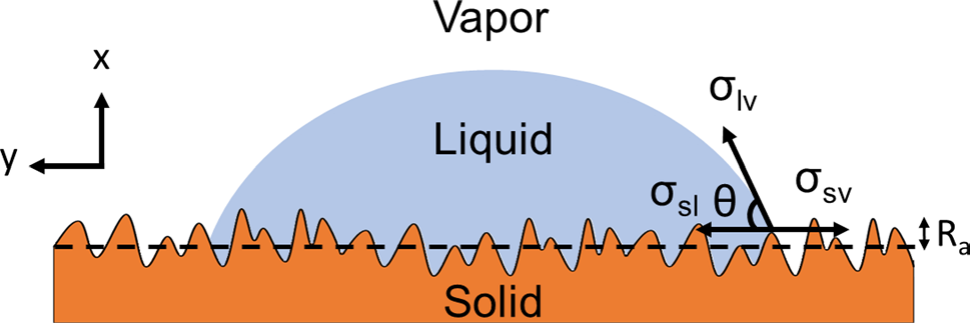
A schematic of a droplet on a flat surface showing three forces of surface tension leading to Young’s equation under static equilibrium on a rough surface.

Considering the above-mentioned assumptions and Fig. 2, Young’s equation can be written as^16,51^:

1 $$\sigma_{sv} = \sigma_{sl} + \sigma_{lv} cos\theta$$

where, *σ* and *θ* are surface tension [N/m] and contact angle [°], respectively. *R*_*a*_ is the average deviation of the surface roughness profile from the mean line [µm]. The contact angle in Eq. (1) is the ideal contact angle on a smooth surface where there is no roughness. To incorporate the effect of surface roughness, Eq. (1) should be modified. Wenzel^40^ proposed a relationship defined by the ratio of the actual surface area of a solid surface to the geometric surface area, or roughness factor, affecting the contact angle:

2 $$r = \frac{actual \;surface\; area}{{geometric \;surface \;area}}$$

Using Eq. (2), the apparent contact angle of a rough surface, $\theta^{*}$, with a roughness factor of *r*, can be related to $\theta$, as follows:

3 $$Cos \theta^{*} = r \; Cos \theta \left( 3 \right)$$

Assuming the droplet size is sufficiently larger than the scale of surface roughness (*v*_*droplet*_ > 0.01 mL), and using Eq. (3), introducing surface roughness leads to a decrease in contact angle on a wetting surface. Equation (1) is then modified to Eq. (4):

4 $$\sigma_{sv} = \sigma_{sl} + r \sigma_{lv} \; cos\theta$$

The change in the interface area [m^2^] between the liquid–vapor and solid–liquid are^23^:

5 $$dA_{lv} = \left( {W + 2R_{a} } \right)dy$$

6 $$dA_{sl} = r\left( {2D + W} \right)dy$$

where, *W* is the micro-groove width [m], *D* is the micro-groove depth [m], *r* is the roughness factor, and *dy* is the infinitesimal change in the capillary height in *y*-direction, see Fig. 1.

The Helmholtz free energy between three interfaces can be written as^52^:

7 $$dE = \sigma_{sv} \; dA_{sv} + \sigma_{sl} \; dA_{sl} + \sigma_{lv} \; dA_{lv}$$

Substituting Eq. (1) in Eq. (7) results in the following:

8 $$dE = \sigma_{lv} \left( {dA_{lv} - r dA_{sl} cos\theta } \right)$$

Since the capillary force is *dE/dy* and with substituting Eqs. (5) and (6) in Eq. (8), the capillary force can be found as:

9 $$F_{c} = \sigma_{lv} \left[ {r^{2} cos\theta \left( {2D + W} \right) - W - 2R_{a} } \right]$$

The subscript “*lv*” is omitted henceforth for simplicity. The capillary force balances the gravity force:

10 $$F_{g} = \rho \; g\; r \left( {D \;W\; h} \right) \; sin\alpha$$

where, *ρ* is the fluid density [kg/m^3^], *g* is gravitational acceleration [m/s^2^], and *h* is the equilibrium capillary height [m]. *α* denotes an inclination angle of the capillary channel [°], e.g., 90° for a vertical micro-groove. Equating Eqs. (9) and (10):

11 $$h = \frac{{\sigma \left[ {r^{2} cos\theta \left( {2D + W} \right) - W - 2R_{a} } \right]}}{\rho grDWsin\alpha }$$

The ideal wetting perimeter and cross-sectional area for a smooth micro-groove are defined as *P*_*w,i*_ = *2D* + *W,* and *A*_*c,i*_ = *D* × *W,* respectively. The actual wetting perimeter and cross-sectional area are given by *P*_*w*_ = *r*(*2D* + *W*), and *A*_*c*_ = *r* × *D* × *W,* respectively.

The capillary height, h, is defined as the macroscopic height of the liquid column, neglecting local variations, corner, or near-wall effects, for simplicity. In other words, this is an averaged capillary rise that satisfies the force balance and accounts for the entire volume of liquid column. It should be noted that for most of the capillary rise calculations, only one capillary height value is needed, not the local variation. For applications where such details are needed, other models should be used.

Non-dimensionalizing Eq. (11), one can conclude:

12 $$h^{*} = \frac{\rho ghsin\alpha }{\sigma } \times \frac{{A_{c} }}{{P_{w} }} = r cos\theta - \frac{{W + 2R_{a} }}{{rP_{w,i} }} = r cos\theta - {\mathcal{L}}$$

where, h^*^ is defined as the non-dimensional capillary height, *rcosθ* is the product of roughness factor and cosine of the ideal contact angle, as described in Eq. (3), and

13 $${\mathcal{L}} = \frac{{W + 2R_{a} }}{{rP_{w,i} }}$$

where, ${\mathcal{L}}$ is a new characteristic length scale defined in this study to include the surface roughness of micro-grooves. The subscript “*i*” is dropped henceforth.

From Eq. (12), a criterion for capillary action to occur can be concluded by setting h^*^ = 0:

14 $$h^{*} = 0 \to r \; cos\theta = \frac{{W + 2R_{a} }}{{rP_{w} }} \quad or\quad r \; cos\theta = {\mathcal{L}}$$

Therefore, for capillary action to occur, h^*^ > *0*, the following should be true:

15 $$W < r^{2} \; P_{w} cos\theta - 2R_{a} \quad or\quad {\mathcal{L}} < r cos\theta$$

Therefore, there is a threshold based on a micro-groove’s width, or characteristic length scale, as defined above, above which capillary action would not occur.

**Figure 3:**
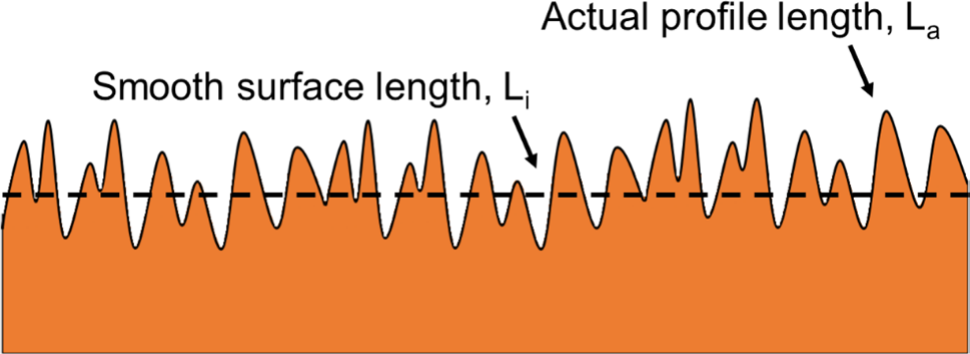
The actual surface roughness profile length vs. the ideal smooth surface length. *R*_*Lo*_ is the percentage increase in the profile length.

Surface roughness can be defined by various parameters and measured by different means. Here, surface roughness is defined based on the EN ISO 21920-2:2021 standard^53^, characterized by *R*_*a*_ and *R*_*Lo*_^54^, and measured by a surface profilometer^55^. *R*_*Lo*_ is the developed length of the surface roughness profile in percentage, as shown schematically in Fig. 3 and is non-dimensional. To determine the roughness factor in Wenzel’s equation, Eq. (3), from surface roughness data, the following is used^56^:

16 $$r = 1 + R_{Lo}$$

Hence, Eq. (12) can be rewritten as:

17 $$h^{*} = cos\theta \left[ {1 + R_{Lo} } \right] - \frac{{W + 2R_{a} }}{{P_{w} \left[ {1 + R_{Lo} } \right]}}$$

Equation (17) is the general form of the capillary rise equation and can be used for any geometry if the geometrical values for *W* and *P*_*w*_ are known. While $P_{w} = 2D + W$ for a rectangular cross section, $P_{w} = 2\sqrt {D^{2} + \frac{{W^{2} }}{4}}$ for a triangular cross-section. A list of different cross-sections, equations for *P*_*w*_, and proof of how the model adapts to different geometries is presented in^13^.

## Experimental study

The surface roughness of a solid surface can be determined by various means, including optical methods, such as laser reflectivity and scanning electron microscopy, and contact methods, such as contact stylus tracing, or a profilometer. Here, a Mitutoyo SJ-400 profilometer was used to measure the surface roughness. The tip of the stylus moves in a line across the surface and measures the peaks and valleys of the solid surface. The stylus height changes vertically over the peaks and valleys. These changes are interpreted internally in the device and a profile is created. This roughness profile is then used to calculate roughness parameters.

**Figure 4:**
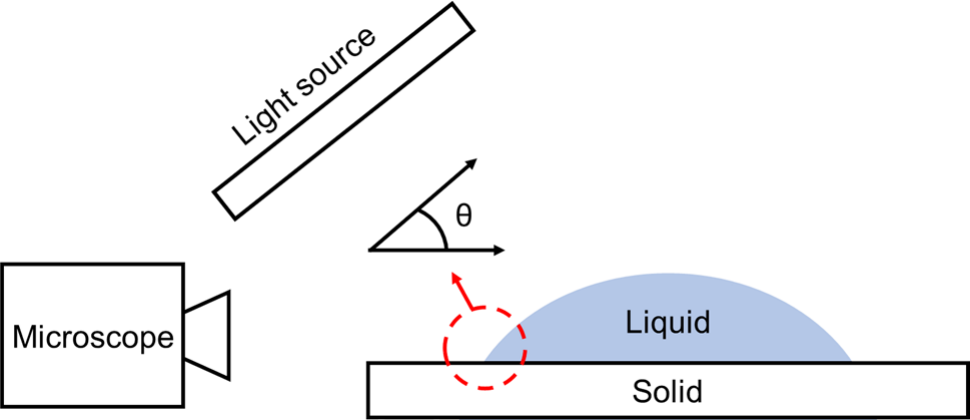
A schematic of the contact angle measurement using the sessile drop method. The contact angle is formed between the liquid–solid–vapor interface.

The contact angle was measured using the sessile drop method. A droplet of water was placed on a flat solid surface. An AM7915MZT Dino-Lite Digital Microscope with ~ 150 × magnification was used for taking images. Image processing was performed with the microscope’s accompanying software. The solid surfaces used were stainless-steel, aluminum, natural graphite sheets, 3D-printed polymer, and 3D-printed stainless-steel. The liquid used in the experiments was water. Measurements were repeated at least five times and an averaged value is reported for the contact angle. The schematic of the test rig used for sessile drop measurements is shown in Fig. 4. Low-power LED lights were used for illumination so that no heat is emitted to the water droplet from the light source. Tests were performed under identical conditions, at room temperature, relative humidity, and pressure. Water droplets were placed on the solid surface gently using a syringe and needle. An approximately 0.05 mL water droplet was used in each measurement^27^.

**Figure 5:**
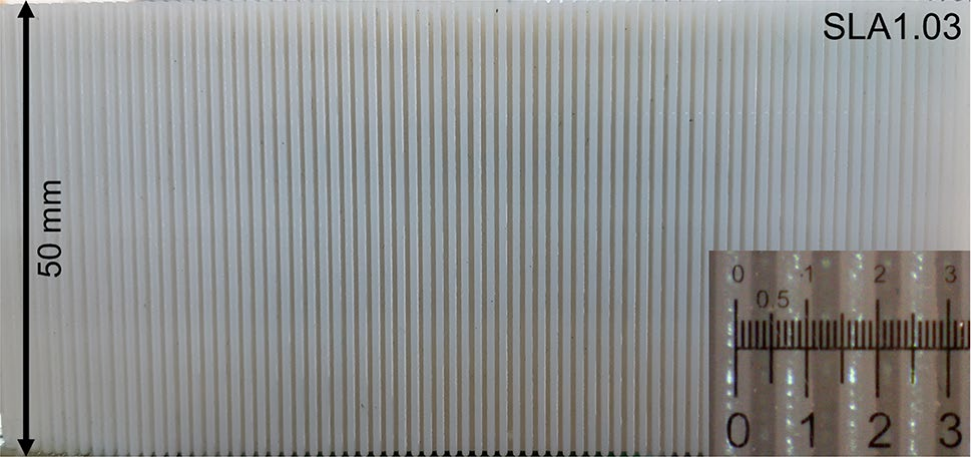
Micro-grooves made by stereolithography (SLA1.03) were used for the capillary rise measurement. The inset shows a micro-groove thickness of 500 µm and a micro-groove width of 400 µm. Micro-groove depth is 1 mm. Table 1 lists the sample details.

In order to experimentally show the capillary rise of water in micro-grooves, various micro-grooves were fabricated. The micro-grooves were created using various methods. Direct Metal Laser Sintering (DMLS) was used for 3D-printed micro-grooves with stainless-steel. Stereolithography (SLA) was used for micro-grooves made with polymers. Figure 5 shows an example of a micro-groove made by stereolithography used for our capillary rise measurements. The inset in Fig. 5 shows a micro-groove thickness of 500 µm, and a micro-groove width of 400 µm, where the micro-groove depth is 1 mm. The micro-grooves were vertically inserted in a container with a pool of liquid water such that a small portion of the micro-grooves were in touch with the liquid. For ease of identifying the capillary height in the images, a 1% by-volume solution of water and food coloring was used. The micro-grooves were left in the pool until the capillary height was steady and the maximum height was achieved. After achieving an equilibrium state, the height of the liquid columns was measured and reported.

Due to the manufacturing defects and variation in surface roughness, the capillary rise in each micro-groove would not be uniform. Therefore, averaged values across the micro-grooves are reported for the capillary height for each sample. Images were taken with the same Dino-Lite Digital Microscope, with ~ 70 × magnification, and at room temperature and pressure. Image processing was performed with the microscope’s accompanying software to measure the capillary height. The microscope and the images were calibrated using a target ruler, provided by the manufacturer.

## Sample preparation

**Figure 6:**
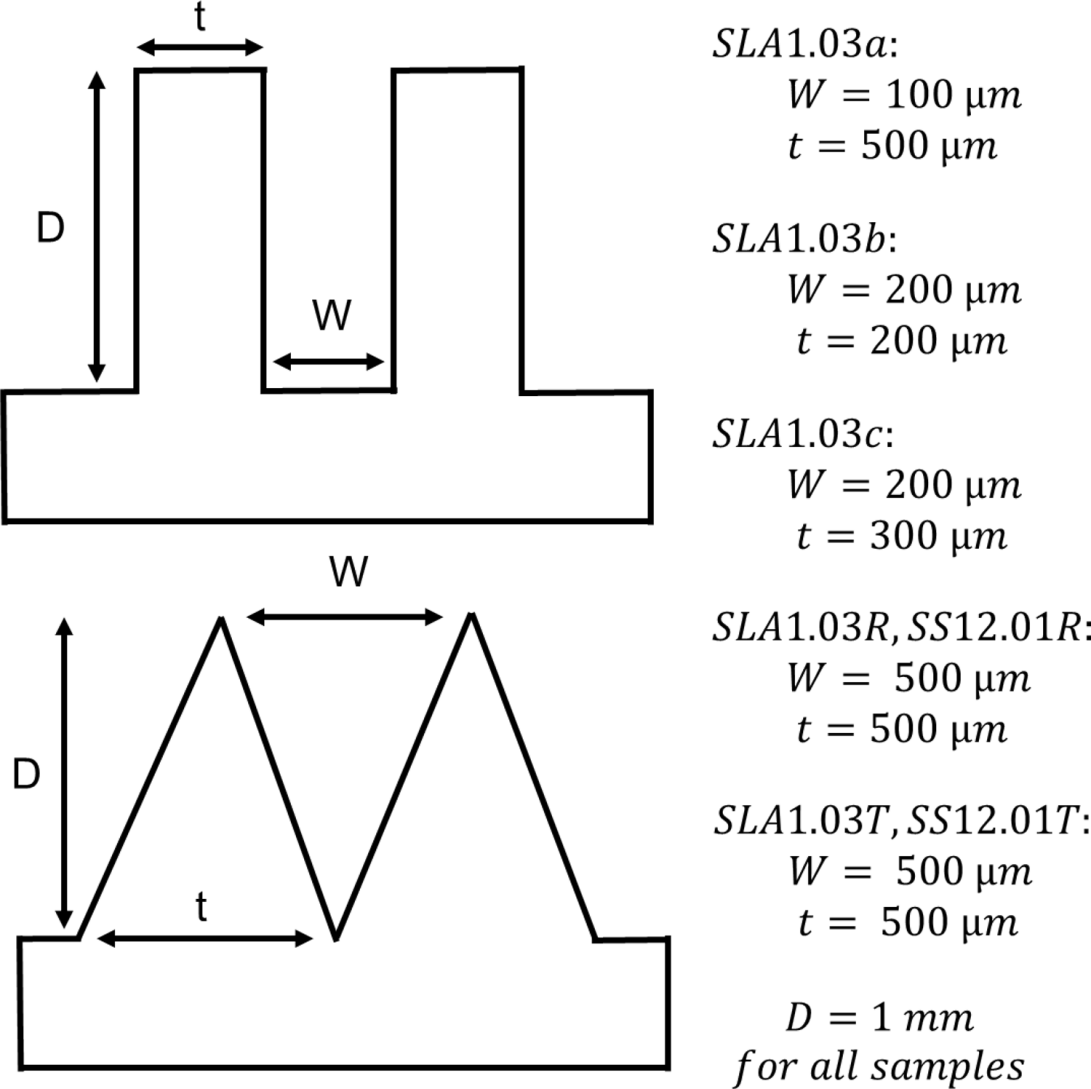
A schematic and dimensions of micro-grooves with various thicknesses and spacing fabricated to measure the capillary height. *SLA* stereolithography, *SS* stainless steel (DMLS), *R* rectangular, *T* triangular.

**Figure 7:**
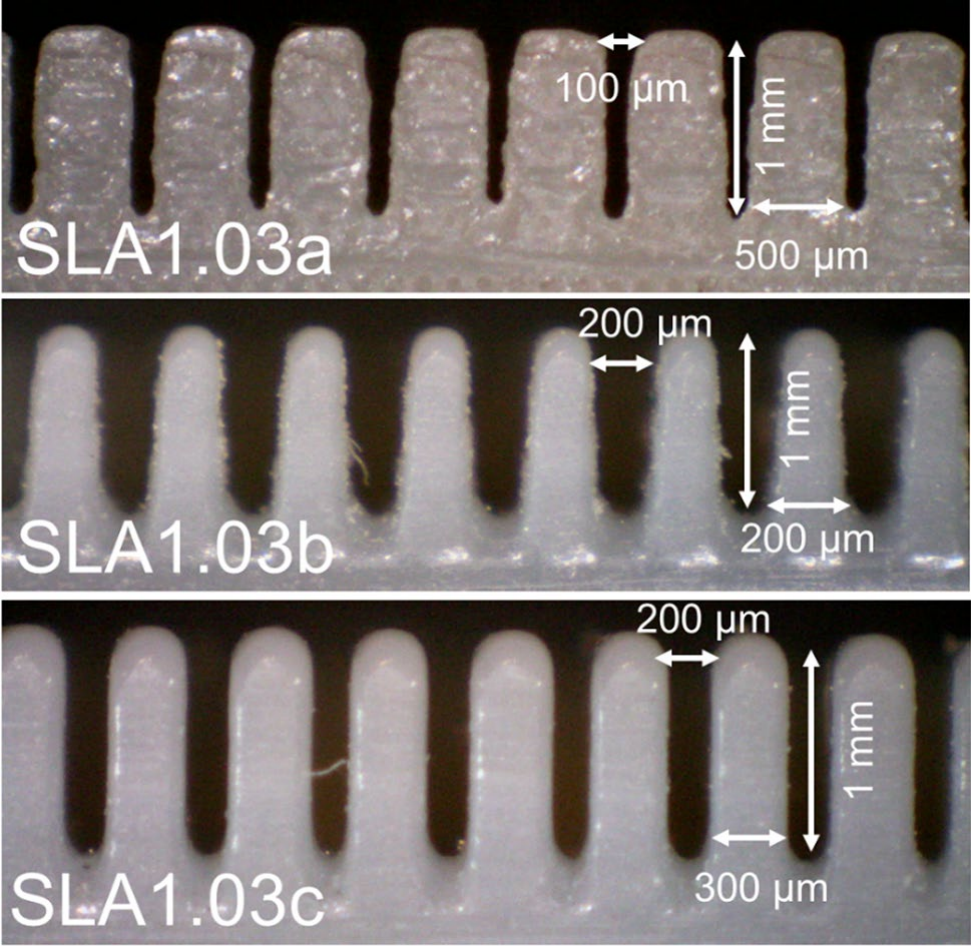
SLA (stereolithography) micro-grooves with various thicknesses and spacing fabricated to measure the capillary height. The micro-groove width range is between 100 to 500 µm. The micro-groove depth is 1 mm and their thickness ranges between 200 to 500 µm. Table 1 lists the sample details.

**Figure 8:**
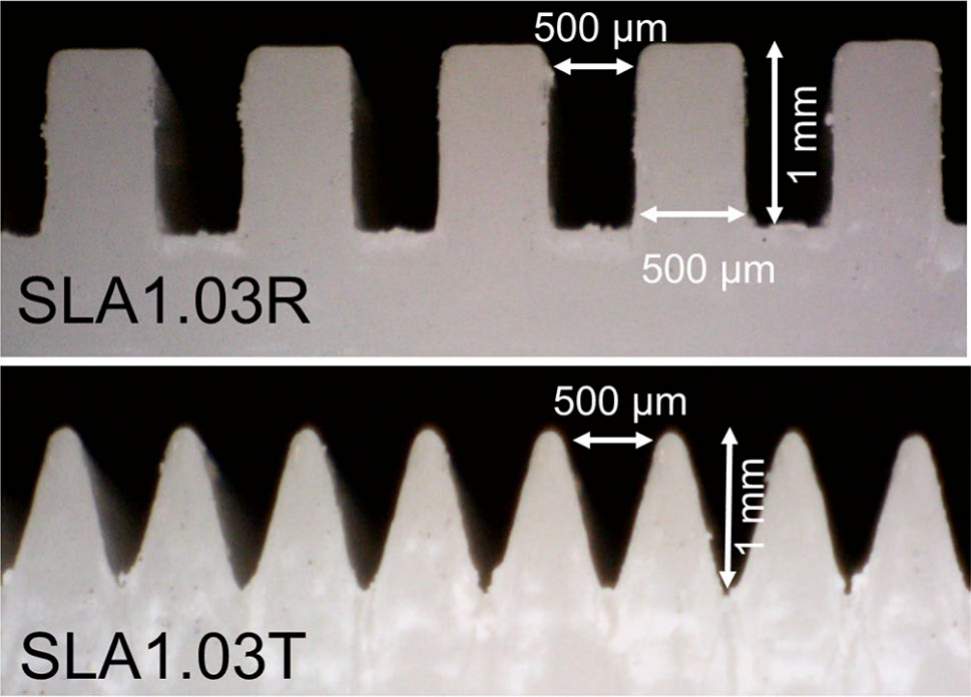
Two cross-sectional micro-groove geometries considered, rectangular (SLA1.03R) and triangular (SLA1.03T) cross-sections, made with stereolithography (SLA). The triangular cross-section has a higher capillary height as compared to the rectangular one. Table 1 lists the sample details.

**Figure 9:**
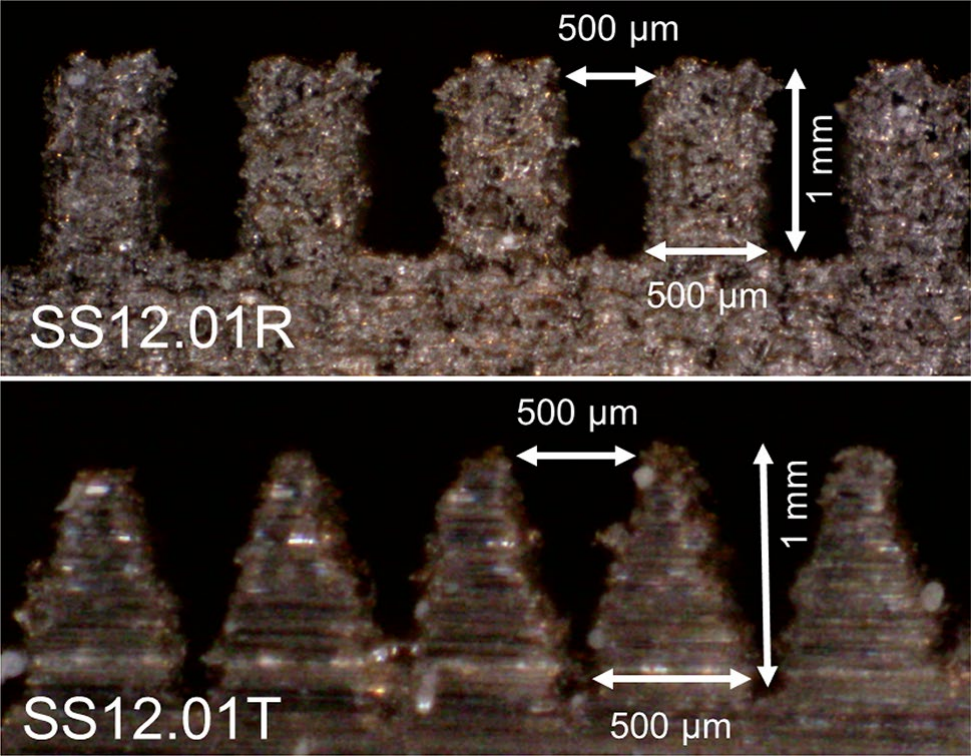
Two micro-grooves considered, rectangular (SS12.01R) and triangular (SS12.01T) cross-sections made with direct metal laser sintering (DMLS). The triangular cross-section has a higher capillary height as compared to the rectangular one, see the results. Table 1 lists the sample details.

The solid surfaces used for the roughness measurement were made of stainless-steel, aluminum, 3D-printed stainless-steel, 3D-printed polymer, and cold-stamped natural graphite sheets, with no coatings. Fine-finished stainless-steel and aluminum sheets were purchased and then roughened with abrasive polishing. Micro-grooves with various micro-groove depth and spacing were fabricated to make a comprehensive study. It was shown in our previous study^13^ that as the micro-groove spacing, or micro-groove width, reduces, the capillary height increases. The micro-groove depth has little effect on the capillary height. Figure 6 shows a schematic and dimensions of micro-grooves with various thicknesses and spacings fabricated to measure the capillary height. Figure 7 shows the cross-section of various micro-grooves used for capillary height measurements in the present study. The micro-groove width range is between 100 to 500 µm with a micro-groove depth of 1 mm. The micro-groove thickness does not directly affect the capillary rise but its variation between 200 to 500 µm is studied as a limiting parameter in the fabrication process, and in compactness. As seen in Figs. 8 and 9, aside from variations in micro-groove dimensions, two main cross-sectional geometries were considered, rectangular and triangular micro-grooves. The triangular cross-section is expected to have a higher capillary height as compared to rectangular one, as per our model predication^13^.

## Uncertainty analysis

The accuracy of the roughness measurements was 0.001 µm. The contact angle accuracy was 0.01°. The microscope used for measuring the capillary height was accurate to 0.1 μm. Considering the uncertainty in geometry dimensions, measured contact angle, measured capillary height, and standard deviation of data, the uncertainty of our capillary height measurement is estimated to be 18.17%, based on the method proposed by Moffat^57^.

## Results and discussion

The solid surfaces used for the roughness and contact angle measurement were made of stainless-steel, aluminum, 3D-printed stainless-steel, 3D-printed polymer, and natural graphite sheets. Measurements were repeated at least five times and averaged values were reported. Each solid surface was thoroughly cleaned with ethanol and left to dry before performing sessile drop tests. The natural graphite sheets that were supplied commercially were made by pressing graphite flakes and forming them into sheets and then were cold-stamped to form grooves. For metallic surfaces, flat sheets were used.

**Figure 10:**
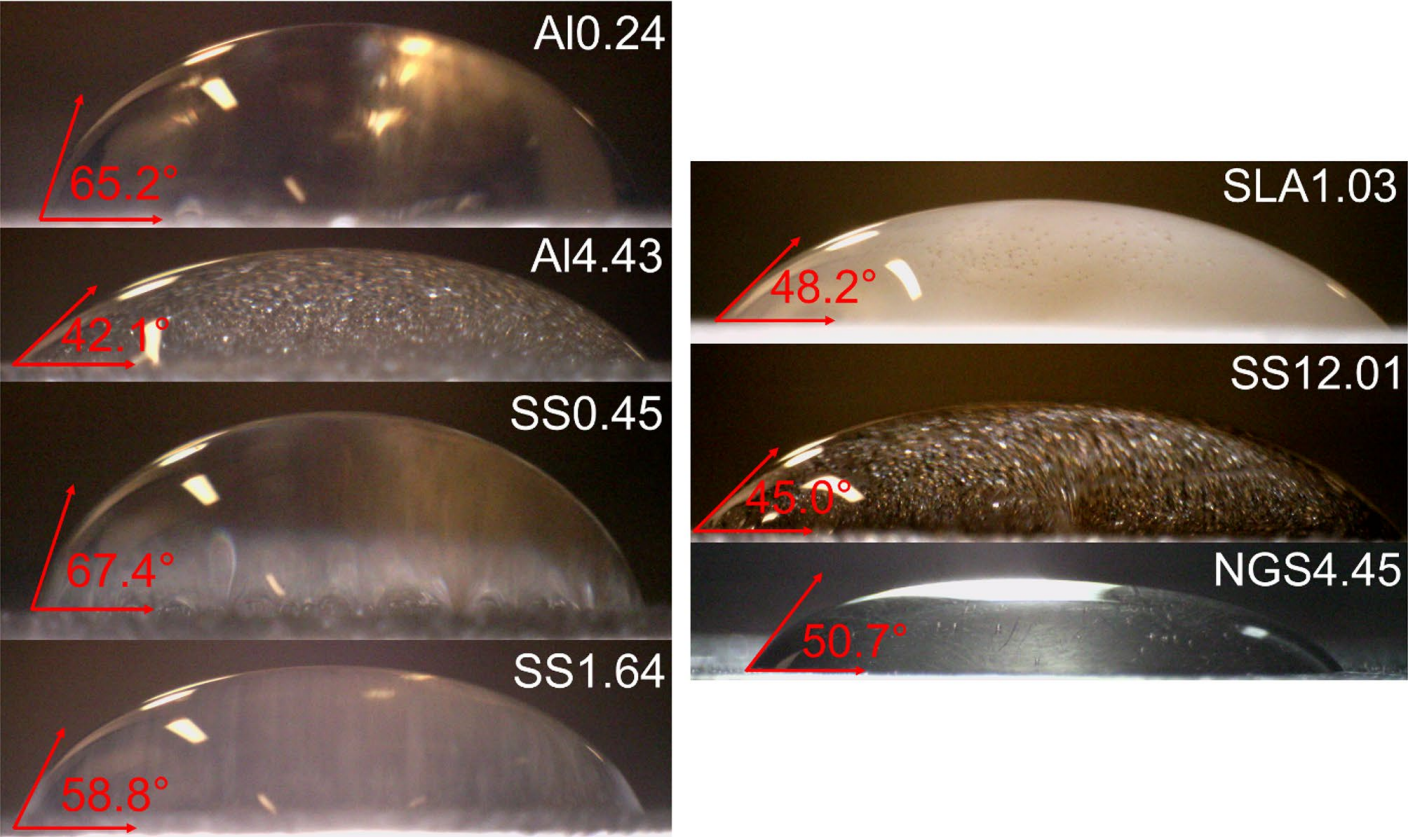
The contact angle measurement using the sessile drop method. Images show the static contact angle formed for water on aluminum, stainless-steel, 3D-printed polymer, 3D-printed stainless-steel, and natural graphite sheet surfaces. Table 1 lists the sample details.

It was observed that with sandblasting, the aluminum sample surface roughness, *R*_*a*_, was increased from 0.24 to 4.43 μm*, R*_*Lo*_ increased from 0.5 to 1.77, and its contact angle decreased from 65.2° to 42.1°. Similarly, the stainless-steel sample surface roughness, *R*_*a*_, was increased from 0.45 to 1.64 μm, *R*_*Lo*_ increased from 0.2 to 0.45, and its contact angle decreased from 67.4° to 58.8°. Since stainless-steel has shown a higher hardness than aluminum, the increase in its surface roughness was less than that of the aluminum sample. The measured surface roughness for the 3D-printed polymer sample was an *R*_*a*_ of 1.03 μm and an *R*_*Lo*_ of 1.21. Its contact angle was measured to be 48.2°. The roughest sample was the 3D-printed stainless-steel with an *R*_*a*_ of 12.01 μm, an *R*_*Lo*_ of 1.12, and a contact angle of 45.0°. The measured surface roughness of the natural graphite sheet, with density of 0.23 g/cm^3^, was an *R*_*a*_ of 4.45 μm, an *R*_*Lo*_ of 0.84, and its contact angle was 50.7°. Figure 10 shows the static contact angle of water on various solid surfaces. As shown in Fig. 10, the decrease in the contact angle of the stainless-steel surface was less that that of the aluminum sample. This can be explained by the fact that the increase in roughness of the stainless-steel sample was less than that of the aluminum sample during the sandblasting process. Table 1 summarizes the surface roughness and contact angle measurement results for the samples.

**Table 1:** A summary of surface roughness values for each sample, measured by the SJ-400 profilometer. Contact angles were measured using a sessile drop method.

Sample	Material	*R*_*a*_ (μm)	*R*_*Lo*_	Contact angle (°)
SS1.64	Stainless-steel	1.64	0.45	58.8
SS0.45	Stainless-steel	0.45	0.20	67.4
Al4.43	Aluminum	4.43	1.77	42.1
Al0.24	Aluminum	0.24	0.50	65.2
SS12.01	3D-printed direct metal laser sintered (DMLS) stainless-steel	12.01	1.12	45.0
SLA1.03	3D-printed stereolithography (SLA) polymer	1.03	1.21	48.2
NGS4.45	Natural graphite sheet (NGS)—0.23 g/cm^3^	4.45	0.84	50.7

**Figure 11:**
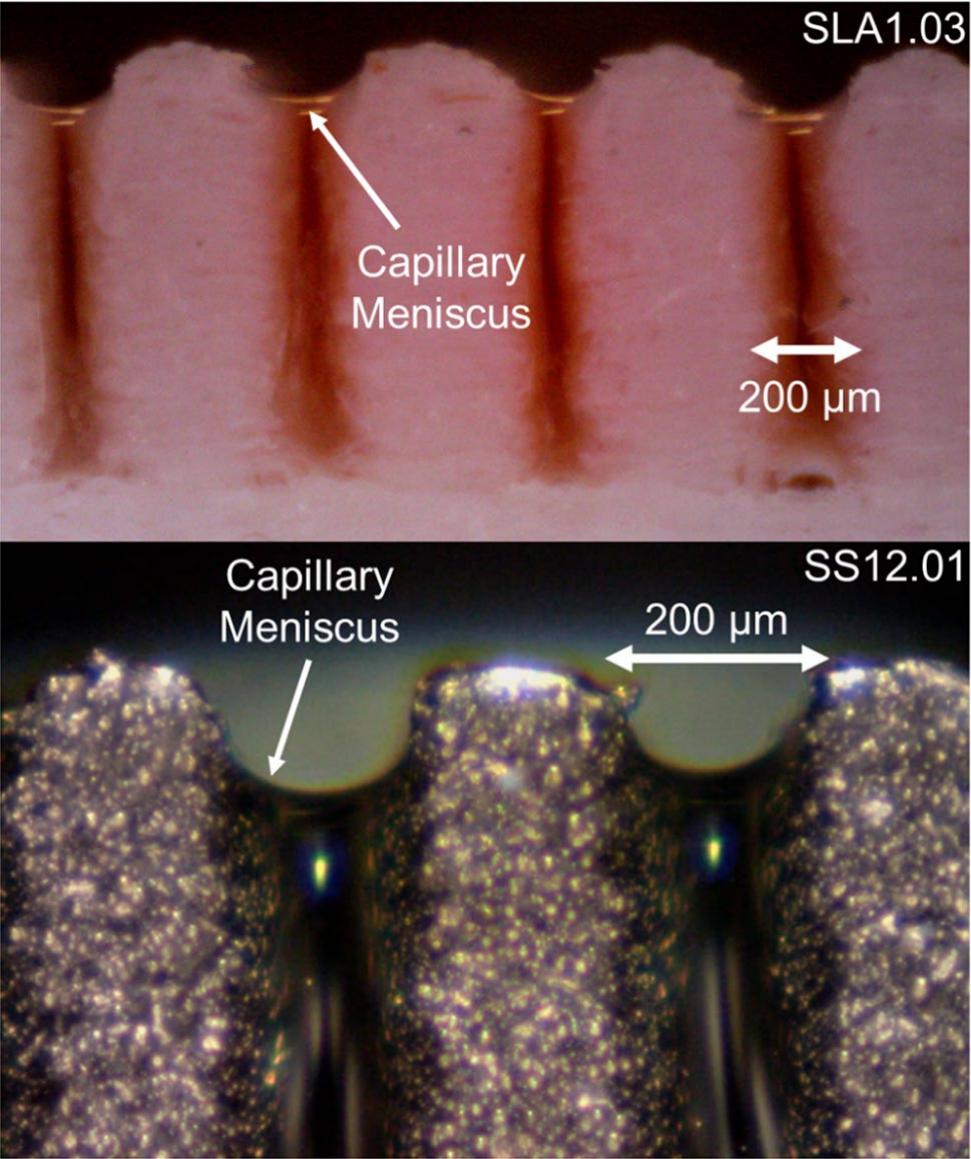
A capillary meniscus is formed between two adjacent micro-grooves due to the disjoining pressure. Top: SLA1.03, Bottom: SS12.01. A 1% solution of water and food coloring is used for better image contrast in the polymer sample. Table 1 lists the sample details.

As shown in Fig. 11, when a liquid comes in contact with a micro-groove, a capillary meniscus is formed between the two adjacent walls due to the disjoining pressure. The capillary meniscus can change based on surface roughness, contact angle, and surface tension. Water properties such as surface tension and density can be found in the literature^58^; at a room temperature of 25 °C; surface tension is 7.28 × 10^–2^ N/m, and density is 999 kg/m^3^.

**Figure 12:**
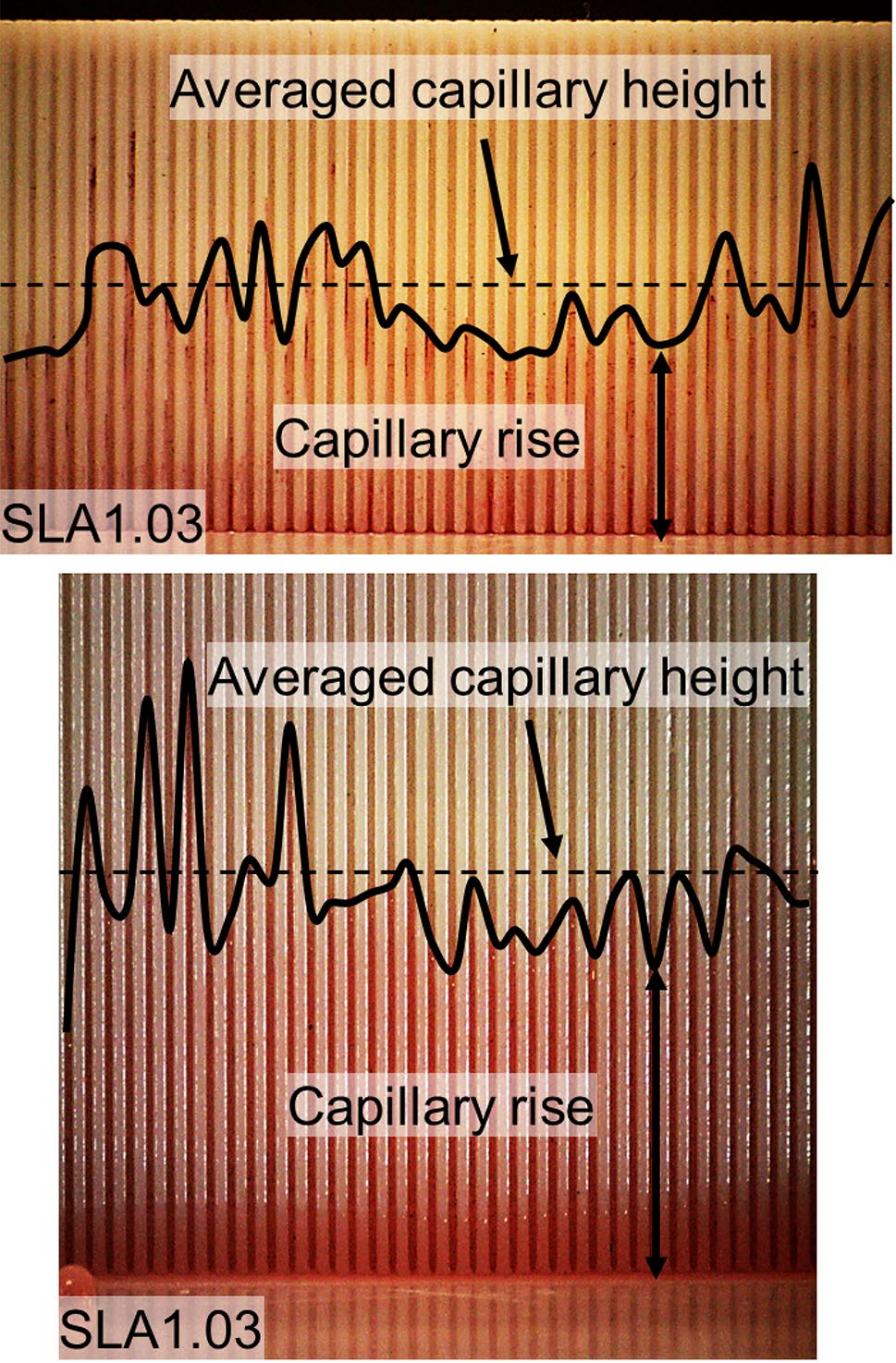
A comparison between the capillary height of SLA1.03 micro-grooves with different micro-groove geometry. Top: rectangular, bottom: triangular micro-grooves. The micro-groove depth is 1 mm, and contact angle is 48.2 degrees. Dashed lines are an average height. Table 1 lists the sample details.

Figure 12 shows the capillary height measurement on two capillary micro-grooves with rectangular and triangular cross-sections. Images were taken by a digital microscope and were analyzed to measure the maximum equilibrium capillary height, as described in section “Experimental study”. The capillary height is not uniform in all micro-grooves due to manufacturing imperfections, variation in dimension, and surface roughness. As an example, the SLA1.03 with a 200-μm micro-groove had an average width of 192 μm, a standard deviation of 0.017, and coefficient of variation of 8%. Therefore, averaged values are reported for capillary height.

**Figure 13:**
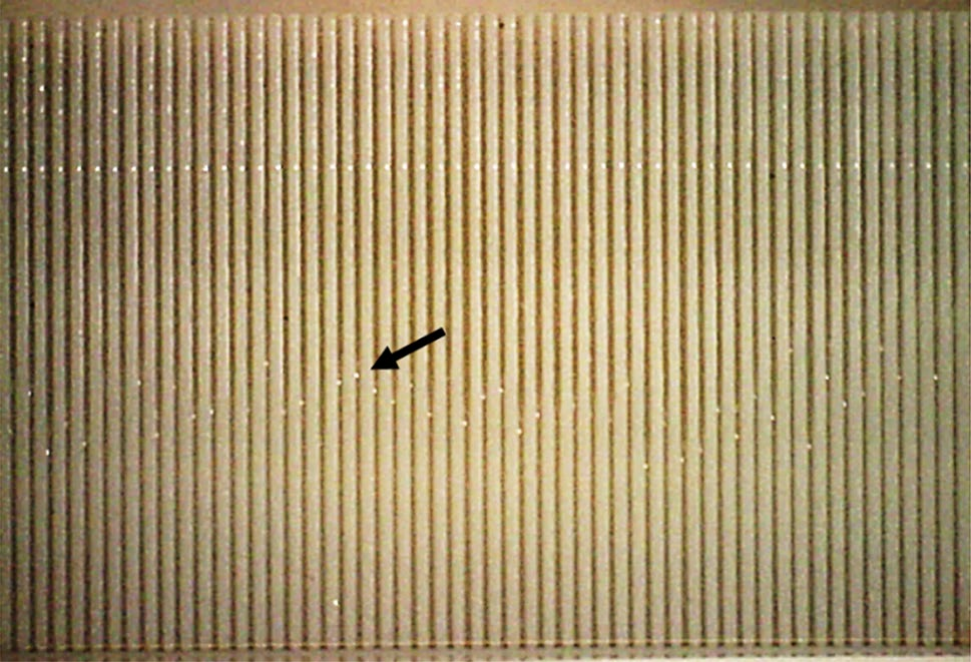
Capillary rise in an SLA1.03 sample without food coloring. The height of the liquid column can be seen as bright spots, by adjusting the light source angle.

It was experimentally verified that the addition of small amount of food coloring (1% solution) has a negligible effect on contact angle and capillary height. Without the food coloring it would be hard to see the capillary rise, as it is shown in Fig. 13. Here, by adjusting the light source angle, the height of the liquid column can be seen as bright spots.

**Figure 14:**
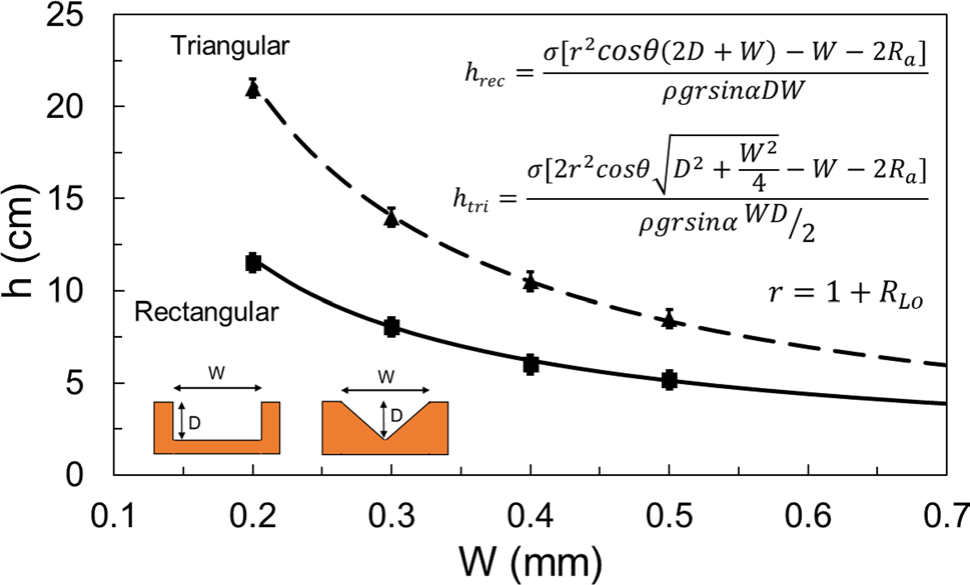
A comparison between capillary height in micro-grooves vs the micro-grooves’ width. Two cross-sections are considered, rectangular and triangular, and experimental results are compared against the present model, Eq. (11). Data for SLA1.03 sample.

Figure 14 shows the predicted capillary height in micro-grooves, using the present model, Eq. (11), versus the measured values for the samples in this study. It can be observed that the capillary height increases rather non-linearly when reducing the micro-grooves’ width. This figure also shows a capillary rise comparison between rectangular and triangular micro-grooves. The experimental results show a good agreement with the present analytical model, Eq. (11), and that discrepancy is within the experimental uncertainties. As previously mentioned, sources of uncertainties include variations in surface roughness, surface oxidization for metallic samples, geometry variations and meniscus corner effects^23^.

**Figure 15:**
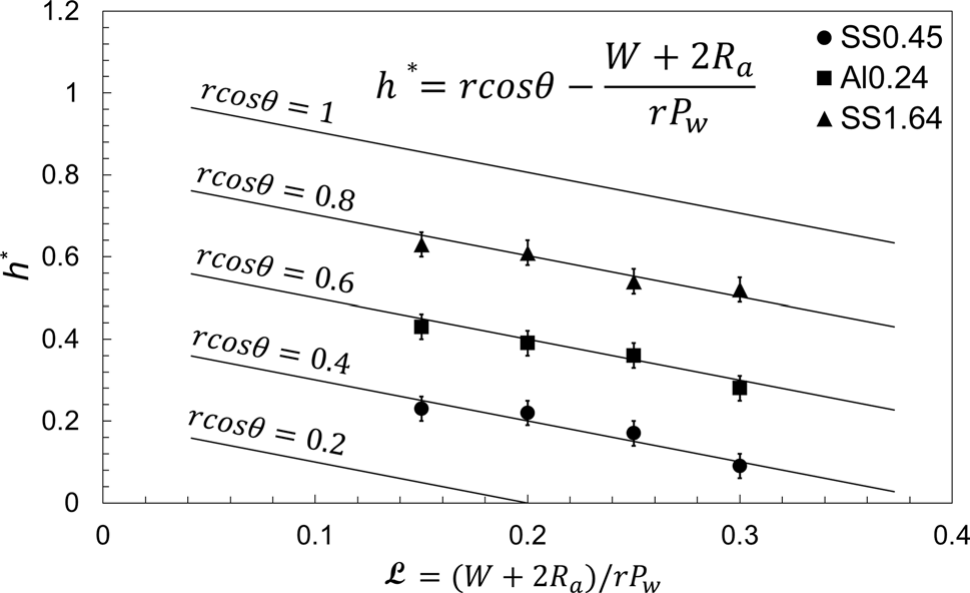
The unifying non-dimensional form of capillary height, Eq. (17), as a function of the characteristic length scale, contact angle, and surface roughness, as compared to our experimental data.

The present unifying model in a non-dimensional form for capillary height is shown in Eq. (17). Figure 15 shows the non-dimensional capillary height, h^*^, as a function of a new characteristic length scale, ${\mathcal{L}}$. For each value of *rcosθ*, the h^*^ is a line that intercepts the x-axis at some point, which is the maximum allowable characteristic length scale for the capillary action to occur. There exists a maximum ${\mathcal{L}}$, for each *rcosθ* value, above which the capillary action would not occur for that *rcosθ*. In other words, for capillary action to occur (h^*^ > 0), the characteristic length scale, ${\mathcal{L}}$, should be smaller than a certain value. This value depends on the micro-groove’s dimensions, contact angle and surface roughness, and equals $r cos\theta$.

**Figure 16:**
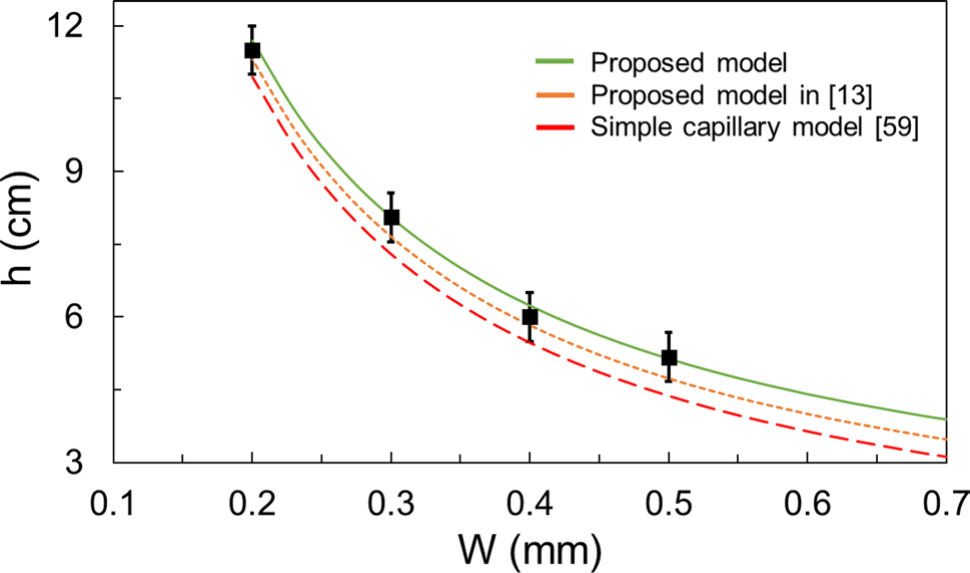
A comparison between capillary height in rectangular micro-grooves vs the micro-grooves’ width. The proposed model is compared to the model in^13^ where surface roughness is not considered in the characteristic length scale (but is considered in the contact angle). Comparison is also made with a simple capillary model based on Young–Laplace equation^59^. Data for SLA1.03 sample.

Figure 16 makes a comparison between capillary height in rectangular SLA1.03 micro-grooves versus the grooves’ width. The proposed model is compared to the model in^13^ where the surface roughness is considered in the contact angle (Wenzel’s contact angle) but not in the characteristic length scale of the proposed model. Comparison is also made with a simple capillary model based on Young–Laplace equaiton^59^, i.e., $h = \frac{2\sigma cos\theta }{{\rho gd_{h} }}$, with *W* as hydraulic diameter.

As shown, the proposed model produces the most accurate results. Similar results were obtained for triangular grooves. Therefore, the proposed unifying model can be used when there is a need for a model that considers not only the cross section of the groove but also the effect of surface roughness, particularly for micro-grooves where the effect of surface roughness is pronounced.

## Conclusion

A previously proposed unifying, non-dimensional model for capillary rise in micro-grooves was extended to include the effect of surface roughness and was experimentally validated. A new characteristic length scale was proposed that includes all of the key geometrical parameters, i.e., micro-grooves height, width, and surface roughness. Furthermore, it was shown that there exists a characteristic length scale, for each modified contact angle value, above which the capillary action would not occur (${\mathcal{L}} > r cos\theta$). The proposed unifying model can be used for any given micro-groove geometry and condition for designing heat pipes, vapor chambers, and capillary-assisted evaporators as well as biomedical devices.

Various metallic and polymer surfaces were fabricated and prepared, including aluminum, stainless-steel, natural graphite sheet, and 3D-printed stainless-steel and polymer. Experimental validation was performed using a profilometer and sessile drop to measure samples surface roughness and the contact angles, respectively. Results showed that there was a less than a 10% relative difference between the new unifying model and our experimental data. It was observed that a triangular cross-section micro-groove, as opposed to a rectangular one, offers a higher wetted area (and capillary height). Wenzel’s model held true for most experimental data. It was concluded that to increase the capillary height in a micro-groove, the wettability of a solid surface can be increased by decreasing its contact angle by roughening the substrate. This is especially advantageous in applications, where compactness and miniaturization are key.

## Data Availability

All data generated or analysed during this study are included in this published article.

## List of symbols

*A*: Area (m^2^)
*D*: Micro-groove depth (m)
*g*: Gravitational acceleration (m/s^2^)
*h*: Average capillary height (m)
${\mathcal{L}}$: Characteristic length scale (m)
*P*_*w*_: Wetting perimeter (m)
*R*_*a*_: Arithmetical mean roughness (μm)
*R*_*Lo*_: Developed roughness length
*r*: Roughness factor
*W*: Groove width (m)
*α*: Inclination angle (°)
*θ*: Contact angle (°)
*ρ*: Density (kg/m^3^)
*σ*: Surface tension (N/m)
